# Soil acidification in continuously cropped tobacco alters bacterial community structure and diversity via the accumulation of phenolic acids

**DOI:** 10.1038/s41598-019-48611-5

**Published:** 2019-08-29

**Authors:** Yuxiang Bai, Ge Wang, Yadong Cheng, Puyou Shi, Chengcui Yang, Huanwen Yang, Zhaoli Xu

**Affiliations:** 1 Yunnan Agricultural University, Kunming, 650201 China; 2 Yunnan Academy of Tobacco Agricultural Science, Kunming, 650021 China

**Keywords:** Agroecology, Agroecology, Agroecology, Agroecology

## Abstract

Studying the obstacles associated with continuous cropping is necessary for sustainable agricultural production. Phenolic acids play an important role in continuous cropping systems, although their mechanism of action in these systems remains unclear. Using High-performance Liquid Chromatography, we characterized the changes in phenolic acid contents in soils that had been continuously cropped with tobacco for different time periods and evaluated the interactions between soil physicochemical properties, bacterial community structure and diversity, and phenolic acids. Prolonged continuous cropping was associated with a significant increase in the content of phenolic acids and a significant decrease in soil pH and bacterial diversity. A significant negative correlation between pH and phenolic acids content was observed, suggesting that soil acidification potentially leads to the accumulation of phenolic acids. The Mantel test indicated that phenolic acids were positively associated with relative bacterial abundance (*R* = 0.480, *P* < 0.01), signifying that the accumulation of phenolic acids is a potential factor leading to changes in bacterial community structure. Continuous cropping lowered the soil pH, which stimulated phenolic acid accumulation and consequently altered the bacterial community structure and diversity, ultimately impacting tobacco plant growth.

## Introduction

The continuous planting of the same crop or related crops in the same plot every year results in poor plant growth and an increase in pests and diseases^1–3^. Continuous cropping has become widespread in China, with more than 20% of the land displaying the severe negative consequences of continuous cropping, such as reduced crop yields and poor soil health. This has become more severe as cultivated land resources have become scarcer and agriculture has become more profit-driven^4^. The problems associated with continuous cropping have thus attracted increasing attention from scientists.

Long-term continuous cropping results in a series of soil problems^5–7^ that lead to increased crop pests and diseases, and reduced yield and quality^8^. Numerous studies have shown that the effect of continuous cropping on the soil environment mainly includes the following four aspects: deterioration of soil physicochemical properties^8,9^, reduction in enzyme activity^10–12^, accumulation of toxic substances^13–15^, and changes in microbial diversity^16,17^. Although previous studies have thoroughly assessed the above four aspects, research regarding their interactions, especially the relationship between toxic substances and other environmental factors, is still unclear and requires further investigation.

Numerous studies have shown that the deterioration of the soil micro-ecological environment is a primary obstacle in continuous cropping^18^, and the role of toxic substance accumulation has garnered increasing research interest^13–15^. There is substantial evidence that the accumulation of phenolic acids is an important contributor to the issues associated with continuous cropping^19,20^. Previous studies have shown that phenolic acids, including p-hydroxybenzoic acid, vanillic acid, p-coumaric acid, and ferulic acid, are widely present in the soil and inhibit the growth of crops^21,22^. Phenolic acids are released into the soil by plant evaporation, leaching, root secretion, litter, and the decomposition of their residues^23,24^. Their contents are affected by soil physicochemical properties, especially pH^25,26^, while microbes degrade phenolic acids and affect their allelopathy^27^. Phenolic acids, as organic substances, will inevitably affect the community structure and diversity of microorganisms while simultaneously providing a C and N source for the microorganisms. Although many studies have shown that the accumulation of phenolic acids alters the microbial community structure^16^, its specific mechanism of influence is unclear.

Soil microorganisms are important for maintaining soil quality. They regulate the decomposition of plant residues and soil organic matter, the biochemical cycle, and the formation of soil structure^28,29^. Many studies have shown that long-term continuous cropping leads to the transformation of the soil microbial community structure from “bacterial type” to “fungal type”^16^, which destroys the soil microbial community structure, leading to an increase in harmful pathogens and a decrease in beneficial flora, which ultimately leads to poor soil health^17,30^. It is thus necessary that the relationships between soil environmental factors, particularly phenolic acids and soil microorganisms, are studied in continuous cropping systems. Previous studies have shown that when phenolic acids enter the soil, they are not only able to inhibit seed germination and seedling growth^31^, but can also alter the microbial community structure and diversity as a result of their particular properties or by acting as signaling molecules^19^. Phenolic acids can significantly affect the biomass, diversity, and community structure of soil microbes, and selectively increase the specific microbial species in the soil^32,33^. For example, vanillic acid in soybean root exudates had a considerable impact on rhizosphere microbial communities^33^. In addition, phenolic acids can also indirectly stimulate soil-borne pathogenic microorganisms and increase plan morbidity, which is a major factor contributing to their role in continuous cropping^34^. For instance, coumarin in cucumber root exudates can significantly affect rhizosphere soil microbial communities and promote the growth of soil-borne pathogens^35^, while vanillin can significantly affect the community structure and abundance of *Fusarium* and *Trichoderma* in cucumber rhizomes^36^. Based on the above studies, it is evident that phenolic acids are a major factor contributing to the obstacles associated with continuous cropping and should be evaluated further.

In the present study, the changes in physicochemical properties, soil enzyme activities, bacterial diversity, and phenolic acids in soil that had been continuously cropped with tobacco for different numbers of years were assessed. The objectives of this study were to: 1) explore the factors contributing to the variation in environmental characteristics, particularly the phenolic acid contents, in soil that had been continuously cropped for different durations, 2) evaluate whether any correlation between soil physiochemical properties, bacterial abundance, bacterial community structure, and phenolic acids exists, 3) improve our understanding of the contribution of phenolic acids to the soil micro-ecological environment under continuous cropping systems, and (4) assess the impacts of continuously cropped soil on tobacco plant growth.

## Results

### Physiological indexes of the tobacco plants

**Figure 1:**
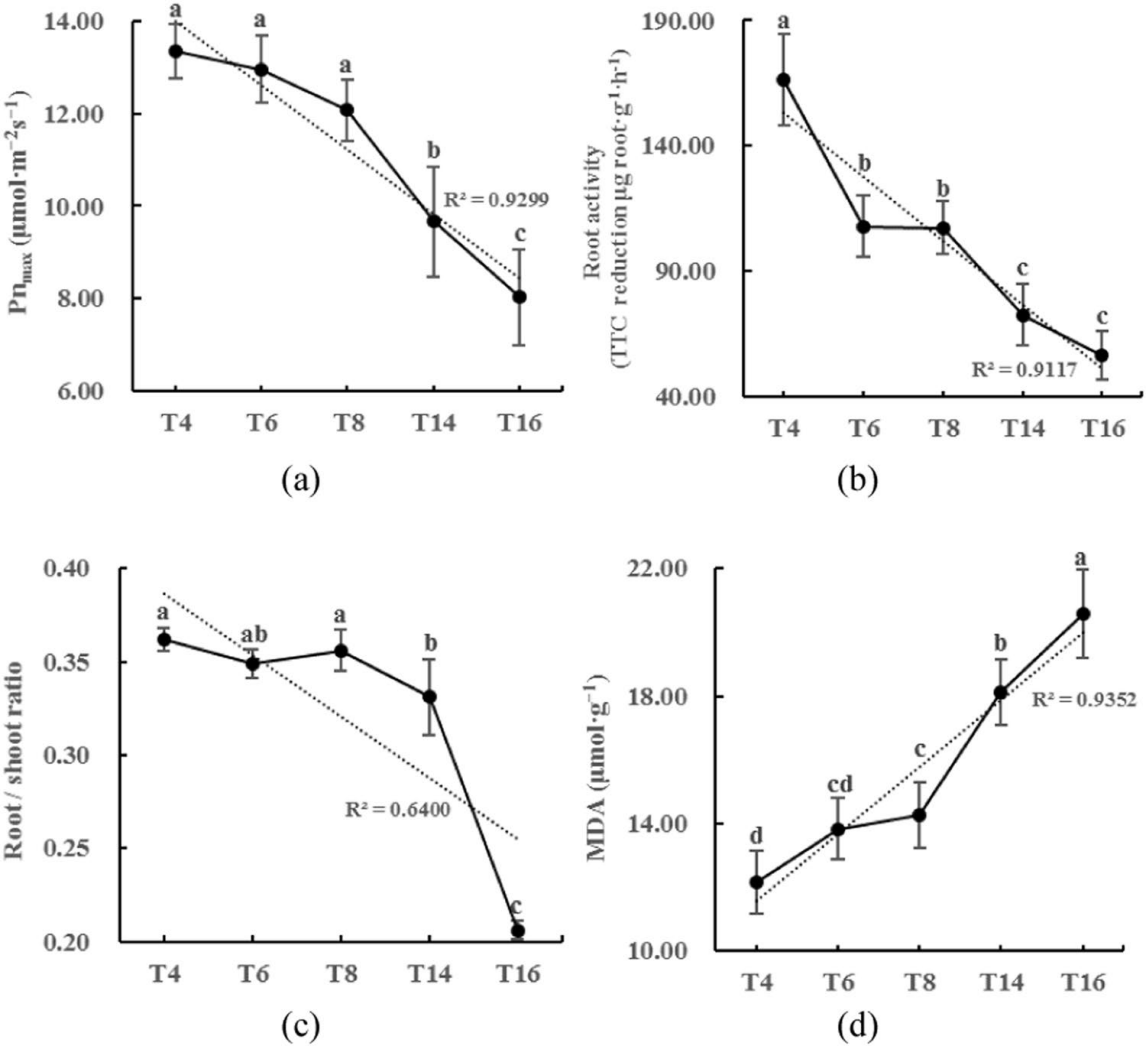
Physiological indicators of the tobacco plants in pots. “T4”, “T6”, “T8”, “T14”, and “T16” represent the five soils planted with tobacco for four, six, eight, 14, and 16 years, respectively. MDA represents malondialdehyde; Pn_max_ represents the maximum net photosynthetic rate. Values are means ± standard deviation (n = 15). Means followed by the same letter for a given factor are not significantly different at *P* < 0.05 (Tukey’s HSD test).

The difference in the maximum net photosynthetic rate (Pn_max_) of the tobacco plants under continuous cropping for 4, 6, and 8 years was not significant; however, it was significantly reduced after continuous cropping for 14 years (Fig. 1a). The Pn_max_ of “T16” was 39.85%, 38.04%, 33.47%, and 16.87% lower than that of the first four treatments, respectively, and the root/shoot ratio also showed the same decreasing trend (Fig. 1b). Although the difference in root activity [measured using triphenyl tetrazolium chloride (TTC)] between “T6” and “T8” and “T14” and “T16” was not significant, the activity of “T4” was significantly higher than the other treatments (Fig. 1c). Conversely, the malondialdehyde (MDA) content of “T16” increased significantly by 69.04%, 48.80%, 44.25%, and 13.42%, respectively, compared with the first four treatments (Fig. 1d).

### Soil physicochemical characteristics and enzyme activities

**Figure 2:**
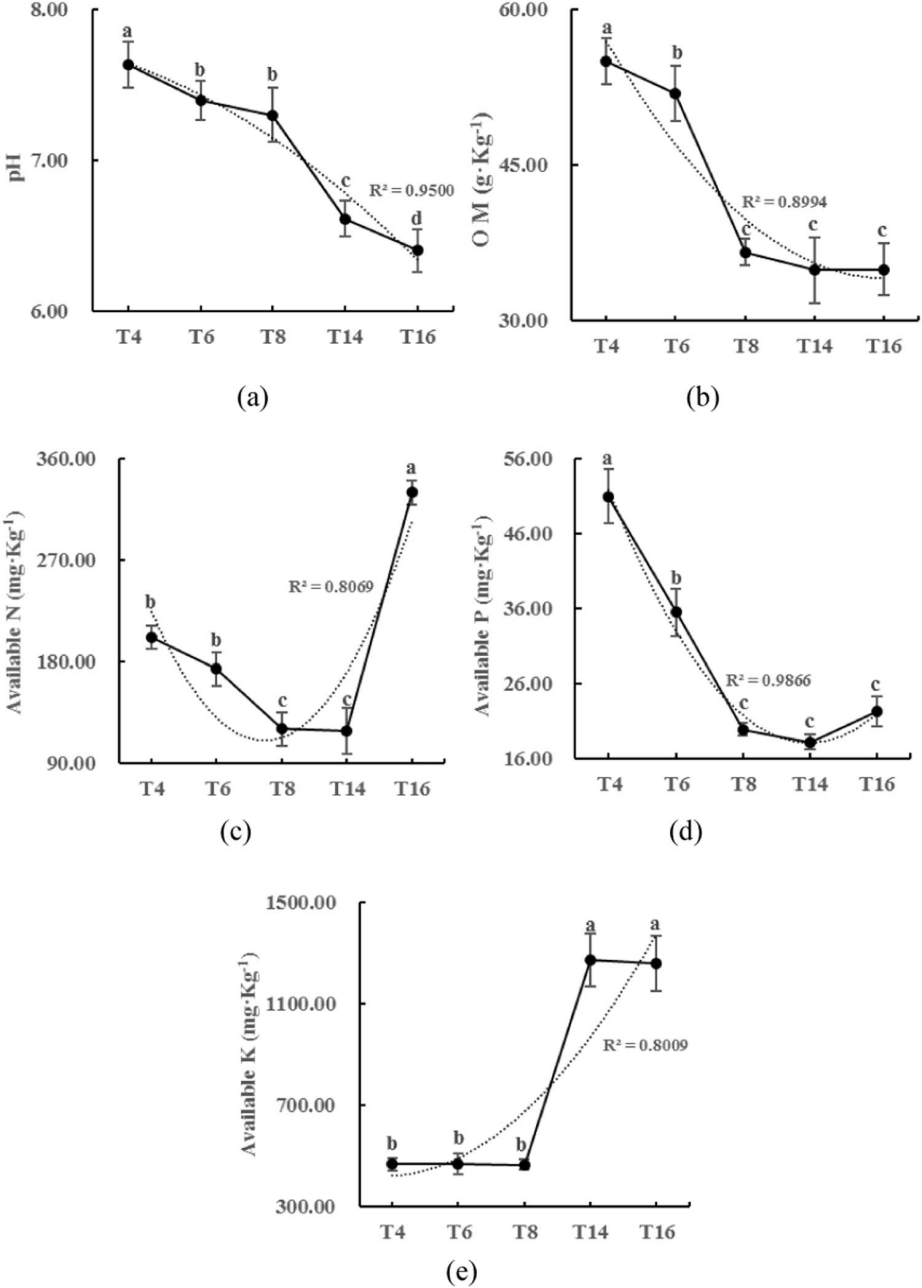
Physicochemical characteristics of the soils from the different sampling sites. “T4”, “T6”, “T8”, “T14”, and “T16” represent the five soils planted with tobacco for four, six, eight, 14, and 16 years, respectively. Values are means ± standard deviation (n = 3). Means followed by the same letter for a given factor are not significantly different at *P* < 0.05 (Tukey’s HSD test).

**Figure 3:**
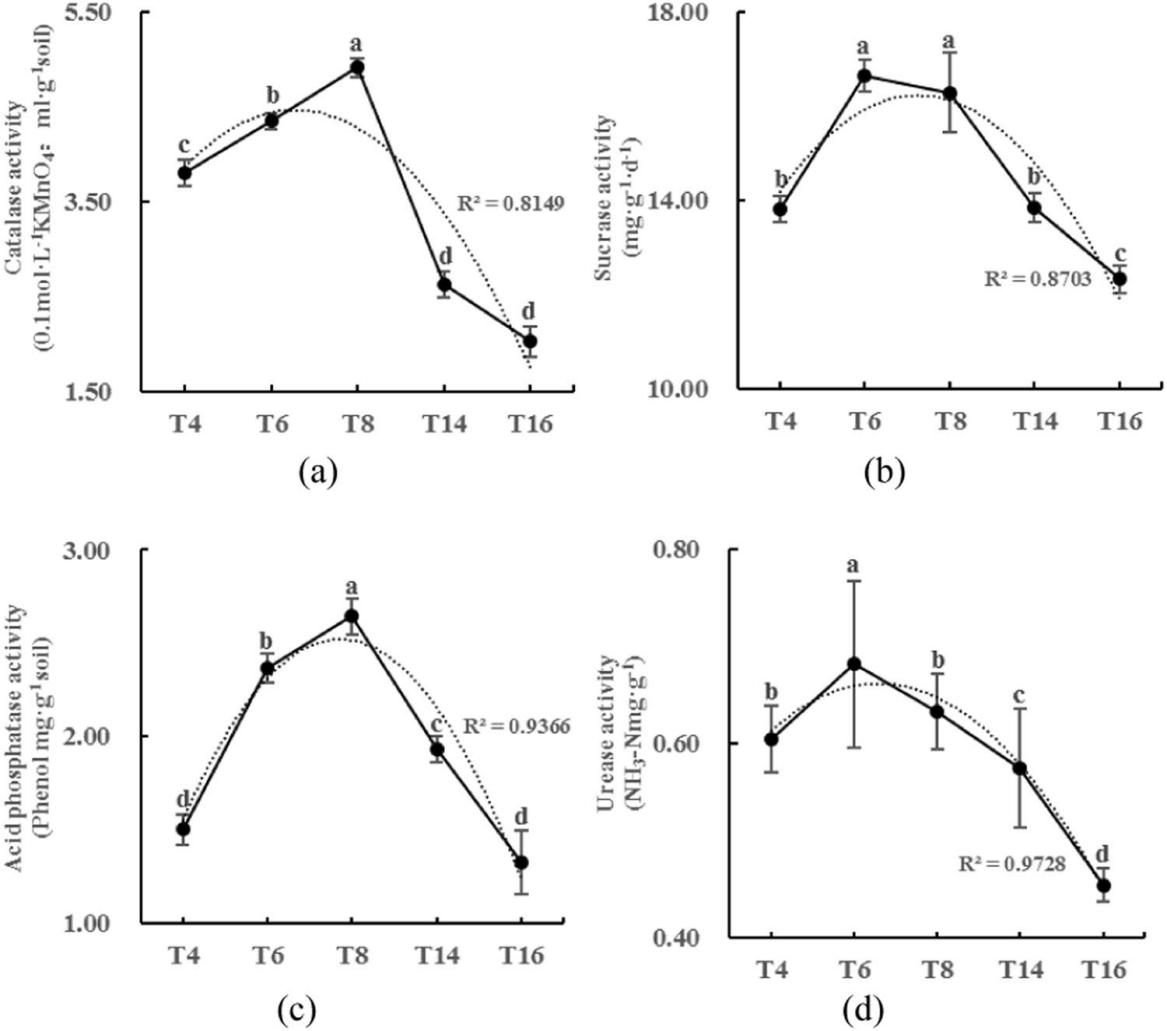
Soil enzyme activities of the soils from the different sampling sites. “T4”, “T6”, “T8”, “T14”, and “T16” represent the five soils planted with tobacco for four, six, eight, 14, and 16 years, respectively. Values are means ± standard deviation (n = 3). Means followed by the same letter for a given factor are not significantly different at *P* < 0.05 (Tukey’s HSD test).

The soil pH, available P, and organic matter (OM) content decreased with the long-term continuous cropping of tobacco (Fig. 2a,b,d), while the available K content increased over time (Fig. 2e). Soil catalase (CAT), urease, acid phosphatase, and invertase activities were assessed to measure the potential turnover rates of N or C in the soils from the five sites. As shown in Fig. 3, the activity of these four enzymes showed a trend of increasing first and then decreasing with the increase in continuous cropping duration. In addition, urease and sucrase activity peaked in the sixth year, while CAT and acid phosphatase activities peaked in the eighth year.

### Phenolic acid contents

**Figure 4:**
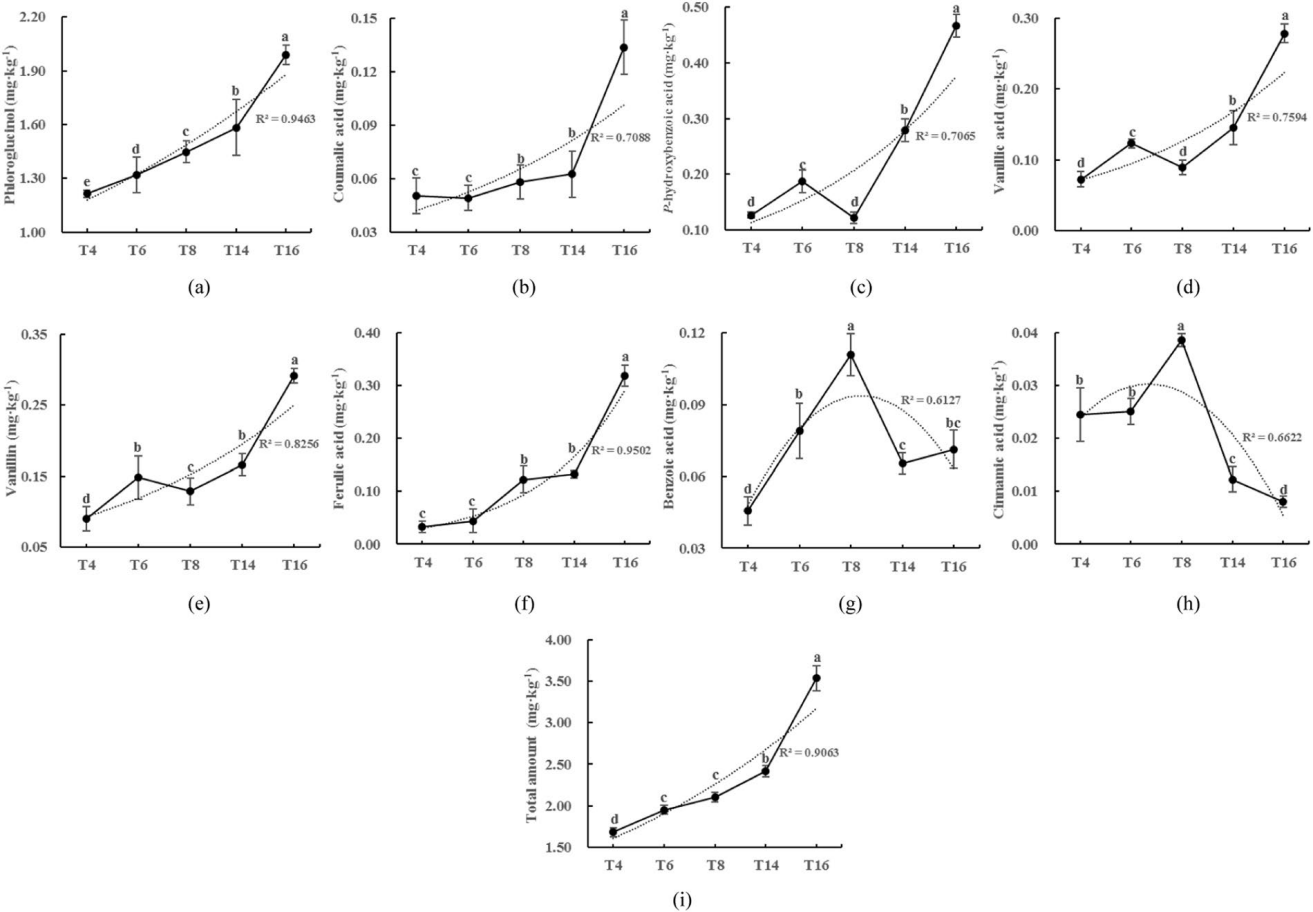
Phenolic acid contents of the soils from the different sampling sites. “T4”, “T6”, “T8”, “T14”, and “T16” represent the five soils planted with tobacco for four, six, eight, 14, and 16 years, respectively. Values are means ± standard deviation (n = 3). Means followed by the same letter for a given factor are not significantly different at *P* < 0.05 (Tukey’s HSD test).

The contents of the phenolic acids in the five soils were measured by High-performance Liquid Chromatography (HPLC). As shown in Fig. 4, the contents of phloroglucinol, coumalic acid, *P*-hydroxybenzoic acid, vanillic acid, ferulic acid, and the total amount increased with the long-term continuous cropping of tobacco. The content of the above phenolic acids in “T16” increased by 61.41%, 168.00%, 267.72%, 280.82%, 223.33%, 893.75%, and 109.92%, respectively, compared with “T4”. In addition, the contents of benzoic acid and cinnamic acid appeared to increase first and then decrease with increasing cropping duration, peaking in the eighth year.

### Bacterial community composition

**Figure 5:**
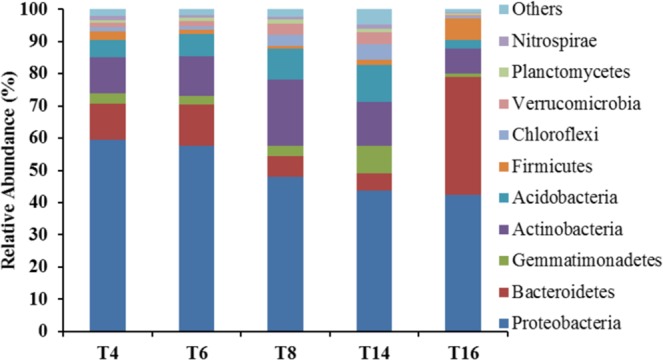
The relative abundances of bacterial phyla in the soils of the five soil sites. “Others” comprised unclassified and low-abundance phyla. “T4”, “T6”, “T8”, “T14”, and “T16” represent the five soils planted with tobacco for four, six, eight, 14, and 16 years, respectively.

**Figure 6:**
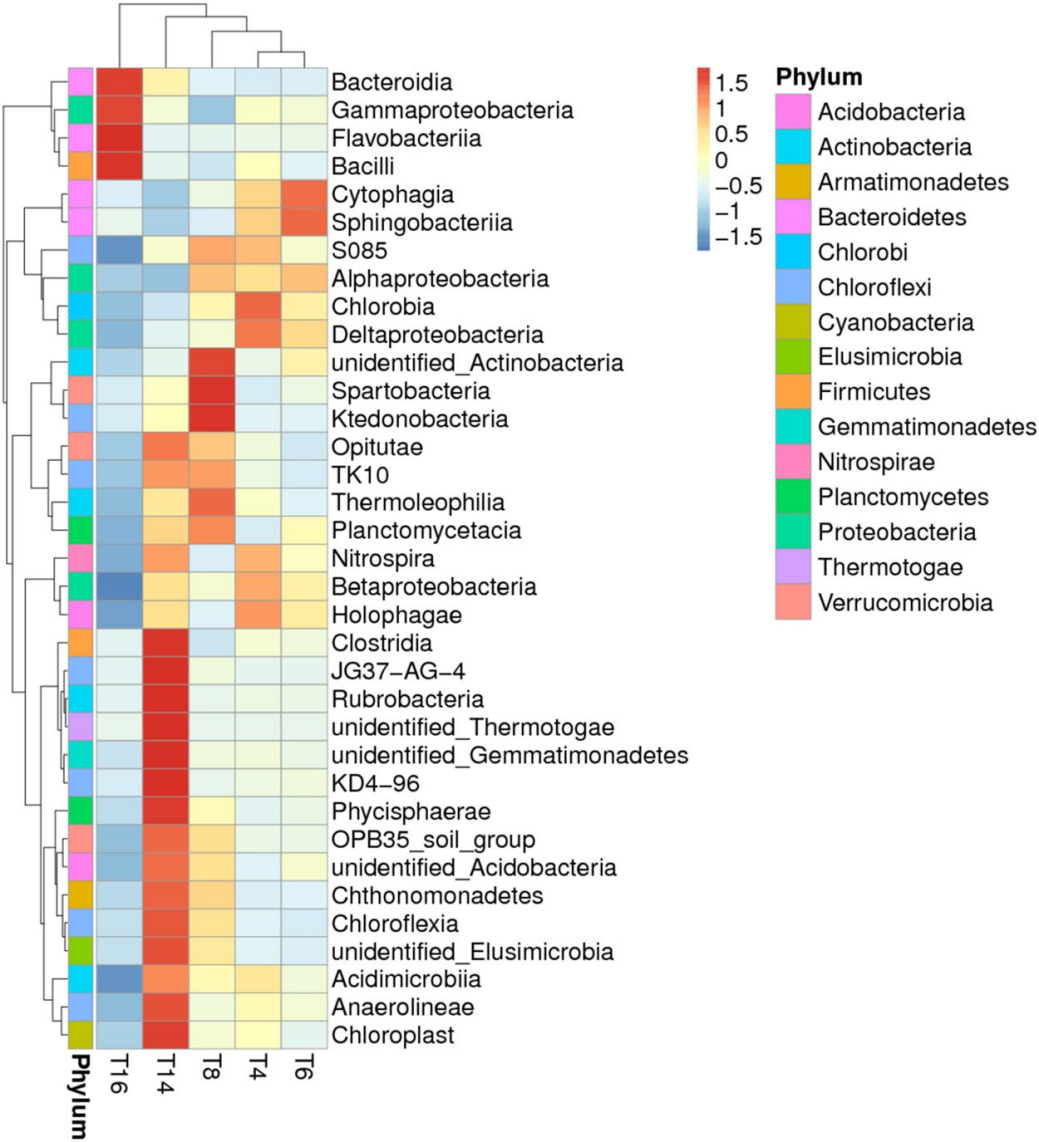
Heatmap analysis of the dominant generic distribution of bacterial communities (top 35 of relative abundance). “T4”, “T6”, “T8”, “T14”, and “T16” represent the five soils planted with tobacco for four, six, eight, 14, and 16 years, respectively. The data were normalized by the Z-score. Blue denotes low relative abundance, while red denotes high relative abundance.

All the sample sequences were classified into 47 phyla using Mothur. The top 10 phyla are shown in Fig. 5. The overall bacterial composition of the different treatments was similar, but the distribution of each phylum varied. *Proteobacteria* (50.27% of all sequence reads), *Actinobacteria* (13.16%), *Bacteroidetes* (14.47%), *Acidobacteria* (7.23%), and *Gemmatimonadetes* (3.64%) were the five most dominant phyla, accounting for 88.76% of the reads. Additionally, the relative abundance of *Proteobacteria* decreased with the long-term continuous cropping of tobacco, while that of *Acidobacteria* and *Actinobacteria* appeared to increase initially and then decrease as the cropping duration increased. The abundance of *Bacteroidetes* first decreased but then increased substantially by T16. Furthermore, comparison of the relative abundances of the top 35 classified bacterial genera showed significant variations among the five sites (Fig. 6), particularly with regards to T16 and the other treatments. The relative abundances of *Chlorobium*, *Deltaproteobacteria*, and *Betaproteobacteria* significantly decreased with increased cropping duration.

### Bacterial alpha diversity

**Figure 7:**
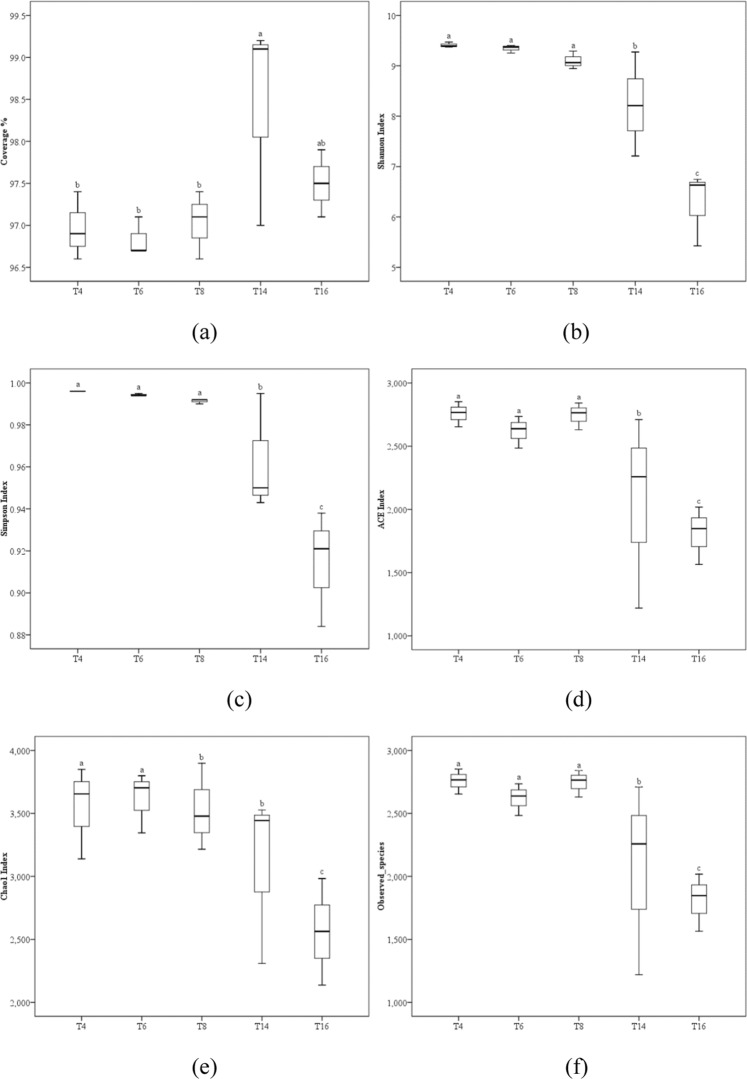
Bacterial α-diversity index of the five soil sites. “T4”, “T6”, “T8”, “T14”, and “T16” represent the five soils planted with tobacco for four, six, eight, 14, and 16 years, respectively. Values are means ± standard deviation (n = 3). Means followed by the same letter for a given factor are not significantly different at *P* < 0.05 (Tukey’s HSD test).

The Good’s coverage values of the five samples were 97.0%, 96.8%, 97.0%, 98.4%, and 97.5%, indicating that sequencing reads were sufficient for this analysis (Fig. 7a). The Shannon, Simpson, ACE, and Chao1 indexes decreased with the duration of cropping, although not all interactions were significant. However, all of the above indexes decreased significantly after 16 years of monoculture (Fig. 7b–e). Furthermore, the number of observed species in the bacterial communities significantly decreased after 14 and 16 years of continuous cropping (Fig. 7f).

### Bacterial beta diversity

**Figure 8:**
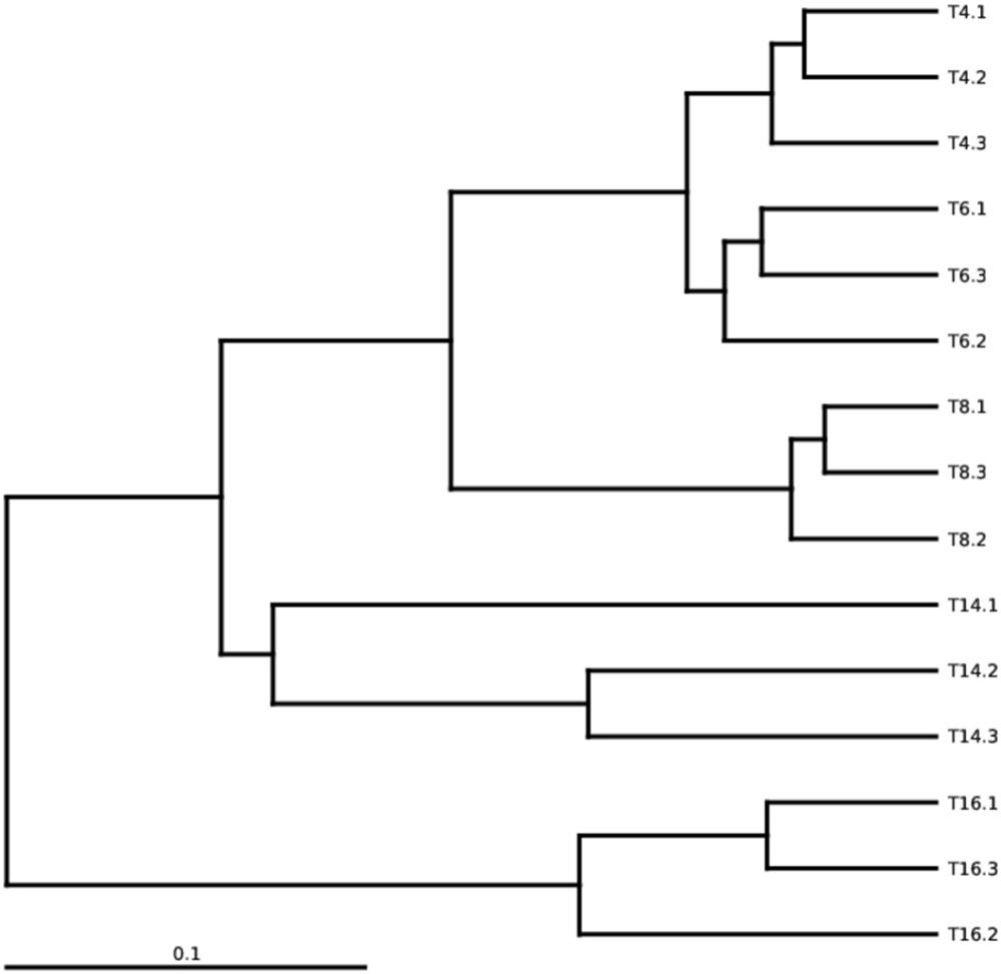
Bray-Curtis dissimilarity hierarchical clustering tree of the soil bacterial communities from the five soil sites. “T4”, “T6”, “T8”, “T14”, and “T16” represent the five soils planted with tobacco for four, six, eight, 14, and 16 years, respectively.

**Figure 9:**
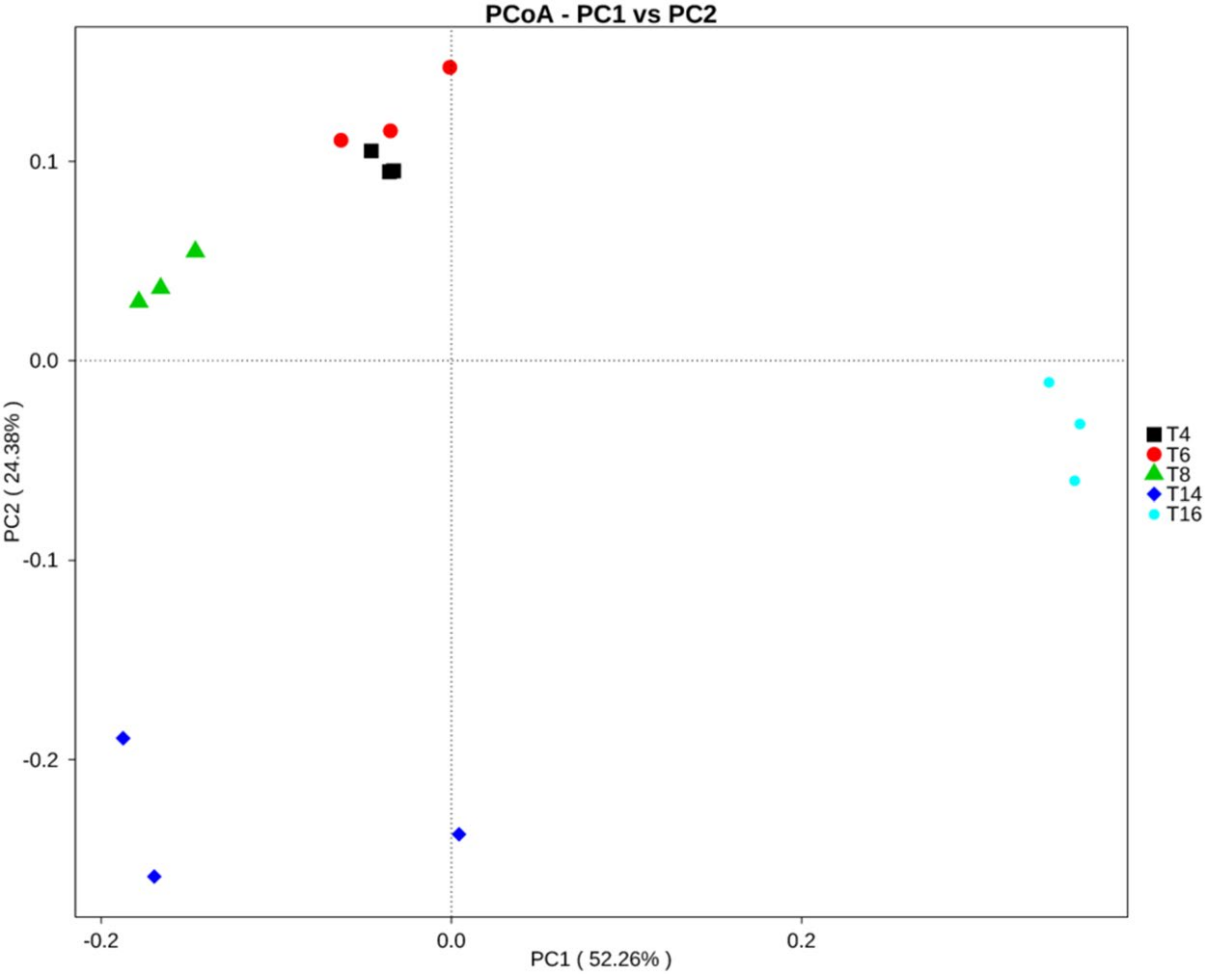
UniFrac-weighted PCoA plots of bacterial communities from the five soil sites. “T4”, “T6”, “T8”, “T14”, and “T16” represent the five soils planted with tobacco for four, six, eight, 14, and 16 years, respectively.

Bray-Curtis dissimilarity hierarchical clustering analysis showed that the samples from the same sites grouped together and were more similar than the samples between different sites (Fig. 8). The “T4”, “T6”, “T8”, and “T14” sites grouped together and were separated from “T16”; the “T4”, “T6”, and “T8” sites grouped together and were separated from “T14”; and “T4” and “T6” grouped together and were separated from “T8”, which suggested that the soil bacterial community structure might be affected by monoculture duration. UniFrac-weighted PCoA based on the operational taxonomic unit (OTU) composition also clearly demonstrated variations among these different soil samples, with the first two axes explaining 52.26% and 24.38% of the total variation for the bacterial data. In addition, the bacterial communities in the four-year soil sample (T4), six-year soil sample (T6), and eight-year soil sample (T8) were obviously separated from the 14-year soil sample (T14) and the 16-year soil sample (T16). Furthermore, the four-year and six-year soil samples had the most similar bacterial community memberships (Fig. 9). The unweighted UniFrac algorithm displayed similar results; for clarity, only the weighted UniFrac PCoA plot is shown here.

### Correlations between soil physicochemical properties and phenolic acids

The Mantel test revealed a significant (r = 0.700, *P* < 0.01) correlation between the soil properties and phenolic acid contents. Spearman’s correlation coefficient was used to assess the relationships between the soil properties and phenolic acids (Table 1). Phloroglucinol, *P*-hydroxybenzoic acid, vanillin, ferulic acid, and total amount were negatively correlated with soil pH. No other significant correlations were observed, with the exception of P-hydroxybenzoic acid and available K. The above results indicate that soil pH and phenolic acid content are closely associated. Furthermore, stepwise regression analysis also demonstrated that pH was the only physicochemical property that was significantly associated with phenolic acid content (Table 2).

**Table 1:** Spearman’s rank correlation coefficients (r) between the soil physicochemical properties and phenolic acids.

	Soil properties	pH	OM	Available N	Available P	Available K
Phenolic acids	0.700**	—	—	—	—	—
Phloroglucinol	—	−0.952*	−0.829	0.544	−0.700	0.844
Coumalic acid	—	−0.856	−0.664	0.762	−0.475	0.746
*P*—hydroxybenzoic acid	—	−0.938*	−0.650	0.683	−0.480	0.890*
Vanillic acid	—	−0.869	−0.589	0.761	−0.447	0.769
Vanillin	—	−0.895*	−0.640	0.706	−0.516	0.790
Ferulic acid	—	−0.890*	−0.805	0.616	−0.636	0.767
Benzoic acid	—	0.024	−0.442	−0.345	−0.605	−0.246
Cinnamic acid	—	0.759	0.307	−0.542	0.135	−0.862
Total amount	—	−0.932*	−0.767	0.682	−0.626	0.821

*Indicates *P* < 0.05; **indicates *P* < 0.01.

**Table 2:** Partial regression equations for the dependent variables selected from the soil physicochemical properties by stepwise regression.

Dependent variables	Partial regression equation	Normalization coefficient	Decisive factor (R^2^)	Significance
Phloroglucinol	P = 5.152–0.516 pH	−0.952	0.907	*P* < 0.05
*P*-hydroxybenzoic acid	PA = 2.137–0.269 pH	−0.938	0.879	*P* < 0.05
Vanillin	V = 1.052–0.125 pH	−0.895	0.801	*P* < 0.05
Ferulic acid	FA = 1.376–0.177 pH	−0.890	0.792	*P* < 0.05
Total amount	TA = 11.261–1.259 pH	−0.932	0.868	*P* < 0.05

P, PA, V, FA, and TA represent phloroglucinol, *P*-hydroxybenzoic acid, vanillin, ferulic acid, and total amount, respectively.

### Correlations between soil physicochemical properties and abundant phyla

**Figure 10:**
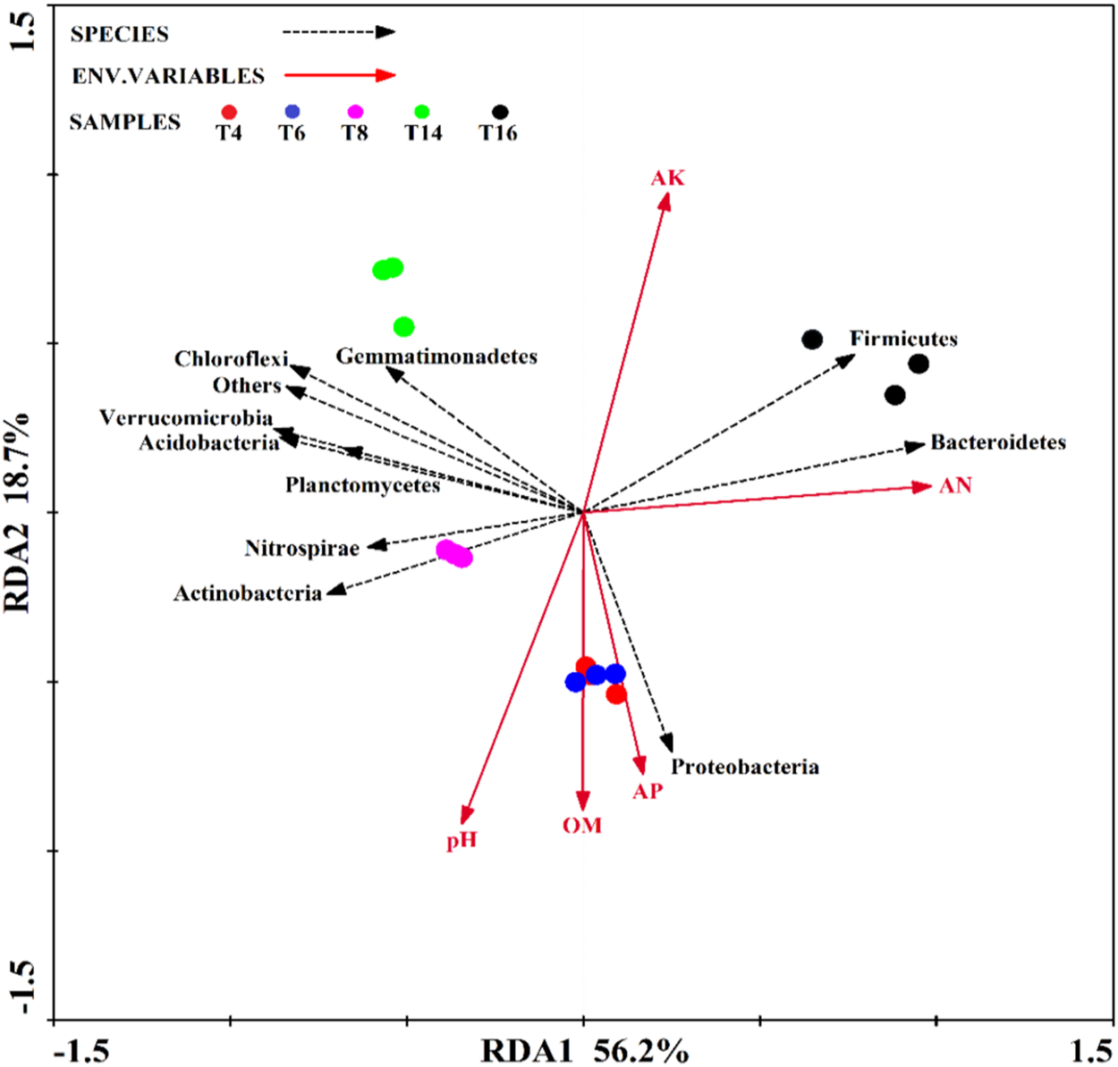
RDA of the abundant bacterial phyla and soil physicochemical properties for individual samples from the five soil sites. “T4”, “T6”, “T8”, “T14”, and “T16” represent the five soils planted with tobacco for four, six, eight, 14, and 16 years, respectively.

The Mantel test analysis revealed a significant (r = 0.440, *P* < 0.01) correlation between the soil properties and the abundance of the bacterial phyla. The first two components of the RDA explained 56.2% and 18.7% of the total variation in bacterial phyla data (Fig. 10). The first component (RDA1) separated “T4” and “T6” from the “T8”, “T14”, and “T16” sites, and the second component also exhibited the same trend (RDA 2). Spearman’s correlation was used to assess the relationships between the soil properties and abundance of the bacterial phyla (Table 3). The abundance of *Proteobacteria* was positively associated with soil OM. The relative abundances of *Bacteroidetes* and *Firmicutes* were positively correlated with soil AN, while the abundances of *Verrucomicrobia*, *Acidobacteria*, and *Planctomycetes* were negatively correlated with soil AN.

**Table 3:** Spearman’s rank correlation coefficients (r) between the abundant bacterial phyla and soil properties.

	Soil properties	pH	OM	AN	AP	AK
Bacterial abundance	0.440**	—	—	—	—	—
Proteobacteria	—	0.781	0.899*	0.243	0.876	−0.790
Bacteroidetes	—	−0.552	−0.241	0.963**	−0.058	0.436
Gemmatimonadetes	—	−0.248	−0.314	−0.623	−0.367	0.459
Actinobacteria	—	0.483	−0.056	−0.809	−0.241	−0.551
Acidobacteria	—	0.047	−0.227	−0.926*	−0.400	0.052
Firmicutes	—	−0.696	−0.352	0.913*	−0.091	0.666
Chloroflexi	—	−0.138	−0.439	−0.825	−0.536	0.230
Verrucomicrobia	—	0.056	−0.351	−0.918*	−0.502	−0.012
Planctomycetes	—	0.075	−0.271	−0.946*	−0.462	−0.016
Nitrospirae	—	0.358	0.426	−0.567	0.395	−0.103
Others	—	−0.059	−0.203	−0.792	−0.292	0.229

*Indicates *P* < 0.05; **indicates *P* < 0.01.

### Correlations between phenolic acids and abundant phyla

**Figure 11:**
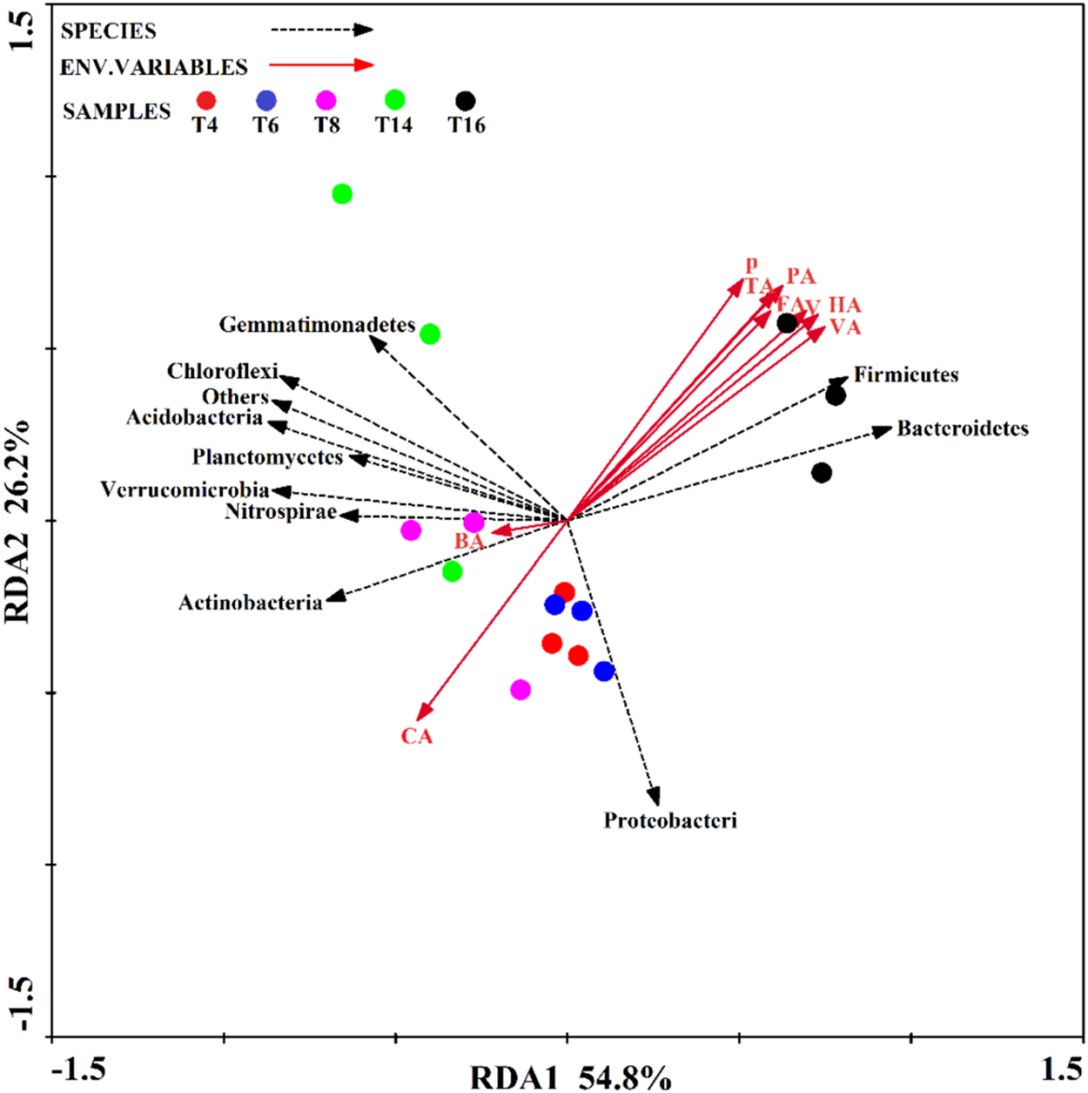
Redundancy analysis (RDA) of the abundant bacterial phyla and soil phenolic acids for individual samples from the five soil sites. “T4”, “T6”, “T8”, “T14”, and “T16” represent the five soils planted with tobacco for four, six, eight, 14, and 16 years, respectively. P, HA, PA, VA, V, FA, BA, CA, and TA represent phloroglucinol, coumalic acid, *P*-hydroxybenzoic acid, vanillic acid, vanillin, ferulic acid, benzoic acid, cinnamic acid, and total amount, respectively.

The Mantel test analysis revealed a significant (r = 0.480, *P* < 0.01) correlation between the soil phenolic acids and the abundance of the bacterial phyla. The first two components of the RDA explained 54.8% and 26.2% of the total variance in the bacterial phyla data (Fig. 11). The first component (RDA1) separated “T4”, “T6”, “T8”, and “T14” from “T16”, and the second component (RDA 2) separated “T4”, “T6”, and “T8” from “T14” and “T16”. Spearman’s correlation was used to assess the relationships between the soil phenolic acids and bacterial phylum abundance (Table 4). The abundances of *Bacteroidetes* and *Firmicutes* were positively correlated with coumalic acid, vanillic acid, vanillin, and total amount. The abundance of *Actinobacteria* was positively associated with cinnamic acid.

**Table 4:** Spearman’s rank correlation coefficients (r) between the abundant bacterial phyla and soil phenolic acids.

	Phenolic acids	P	HA	PA	VA	V	FA	BA	CA	TA
Bacterial abundance	0.480**	—	—	—	—	—	—	—	—	—
Proteobacteria	—	−0.641	−0.411	−0.520	−0.382	−0.441	−0.558	−0.217	0.403	−0.560
Bacteroidetes	—	0.717	0.879*	0.793	0.884*	0.848*	0.768	−0.367	−0.761	0.824*
Gemmatimonadetes	—	−0.005	−0.236	0.009	−0.193	−0.145	−0.137	−0.209	−0.307	−0.083
Actinobacteria	—	−0.458	−0.599	−0.712	−0.693	−0.647	−0.430	0.741	0.871*	0.529
Acidobacteria	—	−0.267	−0.530	−0.378	−0.516	−0.452	−0.375	0.214	0.209	−0.362
Firmicutes	—	0.770	0.908**	0.891	0.891*	0.854*	0.811	−0.367	−0.761	0.824*
Chloroflexi	—	−0.071	−0.331	−0.203	−0.365	−0.302	−0.155	0.237	0.105	−0.173
Verrucomicrobia	—	−0.210	−0.465	−0.398	−0.512	−0.446	−0.273	0.440	0.375	−0.313
Planctomycetes	—	−0.256	−0.522	−0.415	−0.527	−0.459	−0.348	0.370	0.330	−0.355
Nitrospirae	—	−0.620	−0.687	−0.469	−0.636	−0.637	−0.694	−0.573	−0.068	−0.647
Others	—	−0.213	−0.445	−0.237	−0.427	−0.378	−0.325	−0.090	−0.056	−0.298

*Indicates *P* < 0.05; **indicates *P* < 0.01. P, HA, PA, VA, V, FA, BA, CA, and TA represent phloroglucinol, coumalic acid, *P*-hydroxybenzoic acid, vanillic acid, vanillin, ferulic acid, benzoic acid, cinnamic acid, and total amount, respectively.

## Discussion

Previous field experiment studies have shown that continuous cropping leads to a decrease in tobacco plant height, biomass, and root activity^37^. In this study, in addition to the decrease in growth indicators (Fig. 1a–c), we also found that continuous cropping caused a significant increase in MDA content in the tobacco plants (Fig. 1d). MDA content is representative of the degree of membrane peroxidation in plant cells^38^, and the significant increase in MDA content in this study indicates that cells experience oxidative damage under monoculture stress, which may result in an inhibition of tobacco plant growth. Based on the above analysis, we speculate that continuous cropping leads to the accumulation of toxic substances in the soil (such as phenolic acids), which may cause cell membrane peroxidation via allelopathy^39–41^. Additionally, toxic phenolic acids can also indirectly suppress tobacco plant growth by altering the soil micro-ecological environment^42,43^. Continuous cropping was thus associated with a stressful growth environment for the tobacco plants.

Our results showed that continuous cropping leads to soil acidification and nutrient imbalances as well as changes in enzyme activity, characterized by an initial increase followed by a decrease. Specifically, our findings indicate that continuous cropping results in the acidification of the soil, and the acidified soil provides suitable breeding conditions for pathogens^44^, which is one of the major obstacles associated with continuous cropping. The increase in soil available K content may be due to the excessive quantities of K_2_SO_4_ that are added during cultivation, as tobacco is a K-favoring crop. This excessive K^+^ is exchanged with cations, such as Ca^2+^ and Mg^2+^, which further leads to soil compaction and lower OM contents, which in turn aggravates the issues associated with continuous cropping. Previous studies have reported that continuous cropping may result in decreased enzyme activity^10^, but our study found that as the duration of continuous cropping increased, the activity of the four enzymes (CAT, urease, invertase, acid phosphatase) increased first and then decreased (Fig. 3). One explanation for this trend is that prior to the peak period of enzyme activity, the tobacco plants and root residues in the surface soil accumulate yearly, providing sufficient substrates for the enzymes and thus resulting in increased enzyme activity. Once the continuous cropping exceeds the peak period of enzyme activity, the accumulation of pathogens and toxic substances in the soil eventually leads to a decrease in enzyme activity.

In recent years, increasing numbers of studies have demonstrated that phenolic acids have strong allelopathic activity^45,46^. Although the accumulation of phenolic acids in continuously cropped soil has also been widely confirmed, only 3–4 species have been studied in many reports^20,47,48^. In this study, the contents of phloroglucinol, coumalic acid, p-hydroxybenzoic acid, vanillic acid, vanillin, and ferulic acid accumulated significantly with increased continuous cropping duration. A sharp increase in the contents of these phenolic acids was observed after 14 years of continuous cropping, which may be due to the deterioration of the microbial community structure, which resulted in the slow degradation of phenolic acids, eventually leading to their accumulation. Interestingly, we found that benzoic acid and cinnamic acid first increased and then decreased with the increase in continuous cropping duration, peaking after eight years. It may be that benzoic acid and cinnamic acid accumulate at the initial stage of continuous cropping, and as the duration of the continuous cropping increases, the microorganisms that degrade these two phenolic acids become dominant bacteria. This finding has theoretical implications for the screening of novel strains that degrade phenolic acids. The observed changes in the total phenolic acid content in the tobacco-planted soils confirm that long-term continuous cropping leads to the accumulation of phenolic acids that have negative consequences for soil health, further verifying that phenolic acids constitute an important obstacle in continuous cropping.

Our results indicate that continuous cropping leads to changes in soil bacterial community structure and reduced bacterial diversity. Sequence analysis revealed that *Proteobacteria*, *Actinobacteria*, *Bacteroidetes*, *Acidobacteria*, and *Gemmatimonadetes* were the most abundant phyla in the five soil samples (Fig. 5), and this result is generally consistent with the results of previous studies^49–52^. However, some differences in the bacterial species and abundance exist between this study and previous studies, which may be due to differences in climate, vegetation, and soil type. The overall trend of our data indicated that the relative abundances of *Proteobacteria*, *Chlorobia*, *Deltaproteobacteria*, and *Betaproteobacteria* decreased significantly with increasing cropping duration. Furthermore, there was a great difference between T16 and the other treatments (Fig. 6), indicating that long-term continuous cropping most significantly contributes to changes in bacterial community structure. As the period of continuous cropping increased, bacterial diversity showed a significant downward trend (Fig. 7), indicating that reduced bacterial diversity is another major contributor to the issues associated with continuous cropping^53,54^. In addition, both hierarchical clustering and PCoA analyses indicated that continuous cropping of tobacco strongly influenced the variation in bacterial community structure. Particularly, the 14-year and 16-year soils differed from the four-year, six-year, and eight-year soils (Figs 8 and 9), indicating that the long-term continuous planting of tobacco appears to result in a shift in the original state of the soil microbial communities.

The Mantel test indicated a certain correlation between soil physicochemical properties and phenolic acids. Specifically, the correlation analysis demonstrated that the contents of phloroglucinol, *P*-hydroxybenzoic acid, vanillin, ferulic acid, and total amount were negatively associated with soil pH (Table 1). Although the study by Whitehead *et al*.^25^ also showed a negative correlation between pH and phenolic acid content, no significant correlation was observed between the pH values of 6–8. In order to further clarify the relationship between the indicators of the physicochemical properties and phenolic acids, we used stepwise regression analysis to eliminate the variables that were unrelated or less influential. The results showed that pH has a certain inhibitory effect on the content of phloroglucinol, *P*-hydroxybenzoic acid, vanillin, ferulic acid, and total amount (Table 2); that is, the lower the pH value, the more obvious the accumulation of phenolic acids. Phenolic acids are introduced into the soil through several pathways, such as root exudation, pollen transmission, and spoilage degradation of the residue^55^. After entering the soil, they are consumed and decomposed as substrates^56^. However, under continuous cropping conditions, soil acidification is caused by the excessive application of acidic chemical fertilizers^57^. The acidic environment provides suitable conditions for the accumulation of phenolic acids^25^, and the accumulation of phenolic acids exacerbates the acidification of the soil, eventually resulting in an undesirable feedback loop. Based on the above analysis, we speculate that continuous cropping leads to soil acidification, which is turn leads to the accumulation of phenolic acids. We thus recommend that the soil pH is adjusted in agricultural production to reduce the accumulation of toxic phenolic acids.

Previous studies showed that the soil microbial community distribution is associated with these types and concentrations of soil phenolic acids and inorganic nutrients^33,58,59^. In this study, the Mantel test showed that phenolic acids and soil physicochemical properties were significantly correlated with bacterial abundance (Tables 3 and 4). Notably, the correlation between phenolic acids (r = 0.480**) and bacterial abundance was higher than the soil physicochemical properties (r = 0.440**). In terms of the correlation between physicochemical properties and bacterial abundance, the abundance of *Proteobacteria* was positively associated with soil OM, while that of *Bacteroidetes* and *Firmicutes* was positively correlated with soil AN. In contrast, the abundances of *Verrucomicrobia*, *Acidobacteria*, and *Planctomycetes* were negatively correlated with soil AN. However, pH was not significantly correlated with bacterial abundance (Fig. 10 and Table 3), suggesting that pH may indirectly affect bacterial community structure by affecting other environmental factors (phenolic acids, for example) in the soil. Although the total phenolic acid content of the soil was found to increase significantly following two years of continuous *Rehmannia glutinosa* cropping, and expression analysis of different proteins in the rhizosphere soil of a continuous cropping system indicated that the accumulation of phenolic acids caused changes in microbial community structure^47,48^, the above studies did not report on the types of phenolic acids detected. In this study, we further clarified the relationship between different phenolic acids and bacterial abundance. Specifically, coumalic acid, vanillic acid, vanillin, and total amount were significantly associated with *Bacteroidetes* and *Firmicutes* (Fig. 11 and Table 4). Phenolic acids can provide microorganisms with a carbon source and energy for growth and reproduction^56^, which leads to changes in the microbial community structure^60,61^. Previous studies have shown that phenol 2,4-di-tert-butylphenol and vanillic acid can selectively enhance the types of certain microorganisms in the soil^33^. In this study, in addition to vanillic acid, we also found that an increase in coumalic acid and vanillin levels could increase the abundance of *Bacteroidetes* and *Firmicutes*. This might be because these two bacteria can selectively use coumalic acid, vanillic acid, and vanillin as substrates for growth and reproduction. Based on the above results, we speculate that the accumulation of phenolic acids alters the community structure of the soil bacteria by altering the relative abundance of *Bacteroidetes* and *Firmicutes*. This leads to a deterioration in the soil micro-ecological environment may due to the proliferation of pathogenic bacteria and the reduction of beneficial bacteria, etc.^33,60,61^, which ultimately indirectly inhibits tobacco plant growth.

In conclusion, continuous cropping resulted in a lowered soil pH, which stimulated the accumulation of phenolic acids and consequently altered the bacterial community structure and diversity, ultimately leading to poor tobacco plant growth. The above results provide a theoretical basis for further understanding the mechanisms associated with the obstacles of continuous cropping. On this basis, in agricultural production, soil pH, OM, and soil microbiota can be improved through various methods, such as the use of quicklime, green manure, and organic fertilizers, which would provide a better micro-ecological environment for crop growth.

## Methods

### Site description and sampling

The experimental site was located at a contiguous flue-cured tobacco cultivation demonstration area in Shidian County, Yunnan Province, China (99°14′E, 24°36′N), which has a tropical subtropical monsoon climate. The mean annual temperature and precipitation in the area are 17 °C and 1120 mm, respectively. Before the tobacco was planted, the area was a paddy field with the same physical and chemical properties, geographical factors, and ecological environment. Once the tobacco was planted, the annual planting varieties, fertilization regime, and management methods remained the same. No major environmental events occurred during this period. The land in this area has been cultivated by the same farmer, and thus the history of the tobacco planting, including the continuous cropping period, could be determined. At all sites, the soil texture is sandy loam and the flue-cured tobacco variety that had been planted was ‘K326’ (*Nicotiana tabacum*). The annual nitrogen (N) application rate (pure N) was 120 kg∙hm^−2^, the phosphorus (P) application rate (P_2_O_5_) was 90 kg∙hm^−2^, and the potassium (K) application rate (K_2_O) was 321 kg∙hm^−2^. The fertilizer was provided by a local tobacco company, and the cultivation management practices were carried out in accordance with local high-quality tobacco production protocols. The soil samples were collected on September 20, 2017, from five tobacco fields with continuous cropping histories of 4, 6, 8, 14, and 16 years, and were marked as “T4”, “T6”, “T8”, “T14”, and “T16,” respectively. The five tobacco fields were each approximately 2000 m^2^ in size. For each field, three biological samples were randomly collected, and each biological sample consisted of a composite of five soil cores randomly collected from the tobacco-planted soil (3.0 cm in diameter, 0–20 cm in depth). After passing through a 2-mm sieve, each soil sample was divided into three subsamples: one portion for the pot experiment, one portion was air-dried for soil characteristics and soil enzyme activity analysis, while the remainder was snap-frozen in liquid nitrogen and stored at −80 °C for the determination of microbes and phenolic acids.

### Pot trials

A pot experiment was performed to evaluate how the soils from the five different cropping treatments impacted the growth and development of tobacco at the seedling stage from 2017 to 2018. The flue-cured tobacco seedling variety used was K326 and had been cultivated by floating seedlings. Each pot (inner diameter 15 cm, height 15 cm) contained 1.0 kg of soil and one flue-cured tobacco seedling. Each treatment was in triplicate (three blocks), and each block had 10 pots. All the pots were randomly placed in a light incubator with an average temperature of 23 °C and an average humidity of 55%. After 30 d, root/shoot ratio, malondialdehyde content (MDA), root activity, and the maximum net photosynthetic rate (Pn_max_) of the flue-cured tobacco plants were measured. Determination of MDA content was by the thiobarbituric acid method. The root activity was measured according to the triphenyl tetrazolium chloride (TTC) method. The net photosynthetic rate was measured using a LI-6400 portable photosynthetic apparatus (Li-COR Inc., USA) with a blue-red light source probe (Li-6400-02B). The photosynthetically active radiant (PAR) was set to 1800 μmol/(m^2^•s), the CO_2_ injection system was set to 400 μmol/mol, the gas flow rate was 500 mmol/s, and the leaf temperature was set to 25 °C. For the above indicators, five seedlings exhibiting uniform growth in each block were selected, and the average value was calculated following the measurements.

### Determination of phenolic acid contents in the field-collected soil

**Figure 12:**
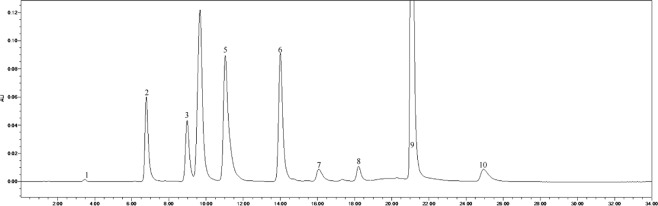
Chromatogram of each standard sample. 1-phloroglucinol; 2-coumalic acid; 3-p-hydroxybenzoic acid; 4-vanillic acid; 5-vanillin; 6-ferulic acid; 7-phthalic acid; 8-benzoic acid; 9-cinnamic acid; 10-salicylic acid.

**Figure 13:**
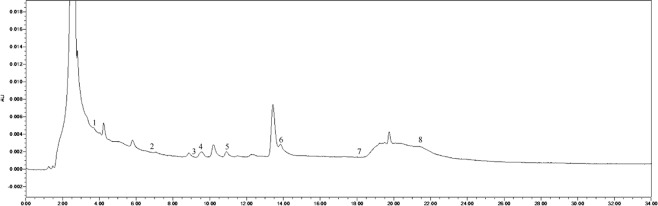
Chromatogram of T4. 1-phloroglucinol; 2-coumalic acid; 3-p-hydroxybenzoic acid; 4-vanillic acid; 5-vanillin; 6-ferulic acid; 7-benzoic acid; 8-cinnamic acid.

**Figure 14:**
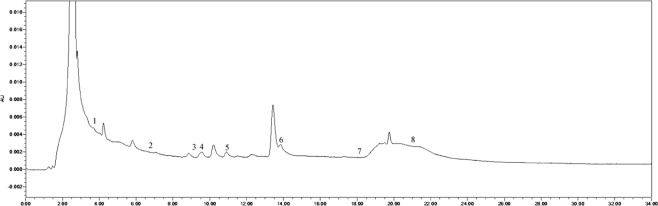
Chromatogram of T6. 1-phloroglucinol; 2-coumalic acid; 3-p-hydroxybenzoic acid; 4-vanillic acid; 5-vanillin; 6-ferulic acid; 7-benzoic acid; 8-cinnamic acid.

**Figure 15:**
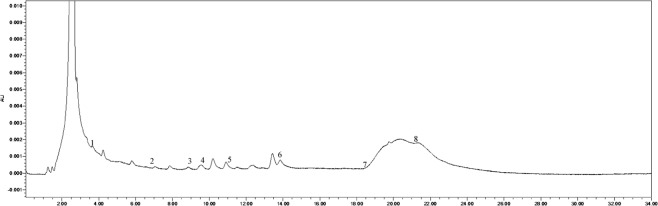
Chromatogram of T8. 1-phloroglucinol; 2-coumalic acid; 3-p-hydroxybenzoic acid; 4-vanillic acid; 5-vanillin; 6-ferulic acid; 7-benzoic acid; 8-cinnamic acid.

**Figure 16:**
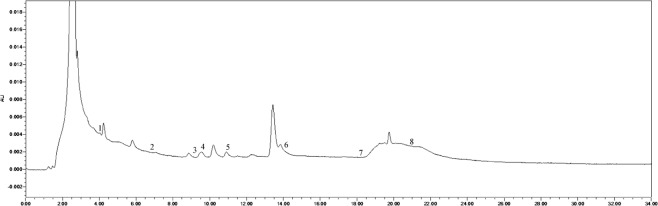
Chromatogram of T14. 1-phloroglucinol; 2-coumalic acid; 3-p-hydroxybenzoic acid; 4-vanillic acid; 5-vanillin; 6-ferulic acid; 7-benzoic acid; 8-cinnamic acid.

**Figure 17:**
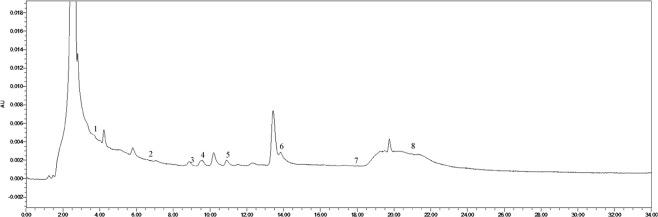
Chromatogram of T16. 1-phloroglucinol; 2-coumalic acid; 3-p-hydroxybenzoic acid; 4-vanillic acid; 5-vanillin; 6-ferulic acid; 7-benzoic acid; 8-cinnamic acid.

The soil phenolic compounds were identified by High-performance Liquid Chromatography (HPLC). One hundred grams of fresh sample was placed into a centrifuge tube (three biological replicates), to which 100 mL of 1 mol∙L^−1^ NaOH was added, allowing for extraction to occur over 24 h. The samples were then shaken for 60 min. Following centrifugation, the solution was acidified to pH 2.5 using 12 mol∙L^−1^ HCl, and after 120 min, the humic acid was removed by centrifugation, following which the supernatant was passed through a fiber membrane of 0.22 μm. The filtrate was then measured by HPLC, and the results were converted according to the weight of the dried soil. The instrument used was an Agilent 1200 (Agilent Technologies, Santa Clara, CA, USA) with a SunFire^TM^ C18 column (4.6 mm × 250 mm, 5 μm) with a flow rate of 1 mL·min^−1^, a column temperature of 25 °C, and a UV detection wavelength of 280 nm. The specific gradients for mobile phase A (methanol) and mobile phase B (aqueous acetic acid solution with pH = 2.5) were: 0 min, mobile phase A 30%, B 70%; 15 min, mobile phase A 50%, B 50%; 16 min, mobile phase A 70%, B is 30%; 30 min, mobile phase A 0%, B is 100%. According to the results of previous studies, 10 phenolic acids were selected as standard samples for determination^21,22^. The standard samples (Sigma) included valproic acid, p-hydroxybenzoic acid, phloroglucinol, vanillic acid, vanillin, ferulic acid, phthalic acid, benzoic acid, cinnamic acid, and salicylic acid, and the injection volume was 10 μL. There was a 10 min delay at the end of each cycle to remove the effects of interfering components and ensure the stability and repeatability of the results. The samples were identified based on the retention time and peak area of the analytical standards. The chromatogram of each standard sample is shown in Figs 12–17.

### Determination of soil physicochemical properties and soil enzyme activities

The soil pH was measured by a potentiometer using a water to soil ratio of 2.5:1 (NY/T 1377-2007). The organic matter (OM) was determined using the potassium dichromate-sulfuric heating method (NY/T 87-1988); available N (AN) was determined by the alkali diffusion method (LY/T 1229-1999); available P (AP) was determined by NaHCO_3_ leaching and the molybdenum antimony colorimetric method (NY/T 1121.7-2006); available K (AK) was determined by ammonium acetate extraction and flame photometer (NY/T 889–2004). The main instruments used for the measurements included a flame photometer, analytical balance, Kay-type nitrogen analyzer, electrothermal constant temperature blast drying oven, and an ultraviolet spectrophotometer. Catalase (CAT) activity was determined by KMnO_4_ titration; urease activity was determined using the indophenol blue colorimetric method; invertase activity was assessed using the 3,5-dinitrosalicylic acid colorimetric method; and acid phosphatase activity was evaluated using the phenylphosphonium phosphate colorimetric method. All the above soil samples were measured in three replicates.

### DNA extraction, 16S rRNA gene amplification, and sequencing

Total genomic DNA from 15 samples (three replicates from each group) was extracted using the cetyltrimethylammonium bromide/sodium dodecyl sulfate (CTAB/SDS) method^62^. DNA concentration and purity were assessed on 1% agarose gels. Based on the concentration, the DNA was diluted to 1 ng/μL using sterile water. The 16S rDNA V4 region was amplified using primers 515 F (5′-GTGCCAGCMGCCGCGG-3′) and 806 R (5′-GGACTACHVGGGTWTCTAAT-3′). All PCR reactions were carried out using Phusion® High-Fidelity PCR Master Mix (New England Biolabs, Ipswich, MA, USA). The same volume of 1 × loading buffer (containing SYB green) was combined with the PCR products and subjected to 2% agarose gel electrophoresis for detection. PCR products was mixed in equimolar ratios. The PCR products were then purified using a GeneJET^TM^ Gel Extraction Kit (Thermo Scientific, Waltham, MA, USA). Sequencing libraries were generated using the Ion Plus Fragment Library Kit 48 rxns (Thermo Scientific) following the manufacturer’s recommendations. The library quality was assessed using a Qubit@ 2.0 fluorometer (Thermo Scientific). Ultimately, the library was sequenced on an Illumina HiSeq2500 platform and 250-bp paired-end reads were generated.

### Sequencing data analysis

#### Paired-end reads quality control

Paired-end reads were assigned to samples using Cutadapt^63^ based on their unique barcode and were truncated by cutting off the barcode and primer sequence. Quality filtering on the raw reads was performed under specific filtering conditions to obtain high-quality clean reads according to the Cutadapt quality-controlled process. The reads were compared with the reference database UCHIME algorithm^64^ to detect chimeric sequences, following which the chimeric sequences were removed^65^, resulting in clean reads.

#### OTU cluster and species annotation

Sequence analysis was performed using Uparse software (Uparse version 7.0.1001)^66^. Sequences with ≥97% similarity were assigned to the same OTUs. Representative sequences for each OTU were then screened for further annotation. For each representative sequence, the Silva Database^67^ was used for taxonomic information based on the Mothur algorithm. To evaluate the phylogenetic relationships of the different OTUs and the differences in the dominant species in the different groups, multiple sequence alignment was conducted using MUSCLE (version 3.8.31)^68^. OTU abundance information was normalized using the standard of the sequence number corresponding to the per sample with the least sequences. Subsequent analysis of alpha diversity and beta diversity were all performed basing on this normalized data.

#### Alpha diversity

Alpha diversity is used for analyzing the complexity of species diversity for a sample via six indices, including Observed-species, Chao1, Shannon, Simpson, ACE, and Good’s coverage. All these indices were calculated for our samples with QIIME (version 1.7.0) and visualized with R software (version 2.15.3).

#### Beta diversity

Beta diversity analysis was used to evaluate the differences in species complexity between the samples. Beta diversity on both weighted and unweighted UniFrac values were calculated using QIIME software (version 1.7.0). Principal component analysis (PCA) was used to reduce the dimensionality of the original variables using the FactoMineR package and ggplot2 package in R software. Principal Coordinate Analysis (PCoA) was performed to obtain the principal coordinates and visualize from complex multidimensional data. A distance matrix of the weighted or unweighted UniFrac values among samples was transformed into a new set of orthogonal axes, by which the maximum variation factor is demonstrated by the first principal coordinate, and the second maximum by the second principal coordinate, and so on. PCoA analysis was displayed using the Weighted Correlation Network Analysis (WGCNA) package, stat packages, and ggplot2 package in R. Unweighted Pair-group Method with Arithmetic Means (UPGMA) hierarchical clustering was used to interpret the distance matrix using average linkage and was conducted in QIIME software.

### Statistical analyses

Using SPSS version 22.0 (SPSS Inc., Armonk, NY, USA) and one-way analysis of variance (ANOVA) with Tukey’s Honest Significant Difference (HSD) tests were conducted for multiple comparisons for all parameters, as were Spearman’s rank correlations and stepwise regression analysis. The correlations between the abundant bacterial phyla and soil characteristics were determined by the Mantel test, and redundancy analysis (RDA) was carried out using the vegan package in R. Heatmap figures were generated using custom R scripts.

## Data Availability

Sequence data were deposited in the Sequence Read Archive (SRA) of the National Center for Biotechnology Information (NCBI) under the accession number SRP186892.
